# Heterologous Expression of Ketoreductase *Ch*KRED20 Mutant in *Pichia pastoris* and Bioreductive Production of (*R*)-1, 3-Butanediol

**DOI:** 10.3390/molecules29184393

**Published:** 2024-09-16

**Authors:** Wanping Chen, Lei Sun, Xinwei Wu, Zhenni Xu, Chin-Yu Chen, Sitong Liu, Haibin Chen, Baoguo Sun, Mingxin Dong

**Affiliations:** 1 School of Pharmacy, Qingdao University, Qingdao 266021, China; wanpingchen@hubu.edu.cn; 2 Enzymaster (Ningbo) Bio-Engineering Co., Ltd., Ningbo 315100, China; lei.sun@enzymaster.com (L.S.); xinwei.wu@enzymaster.com (X.W.); sitong.liu@enzymaster.com (S.L.); haibin.chen@enzymaster.com (H.C.); 3 State Key Laboratory of Biocatalysis and Enzyme Engineering, School of Life Sciences, Hubei University, Wuhan 430062, China; 202221107011792@stu.hubu.edu.cn (Z.X.); chinyuchen@hubu.edu.cn (C.-Y.C.)

**Keywords:** (*R*)-1, 3-BDO, *Ch*KRED20, mutant M12, *P. pastoris*, multi-copy, high-density fermentation, stereoselectivity

## Abstract

(*R*)-1, 3-Butanediol (1, 3-BDO) is an important intermediate in the synthesis of aromatics, pheromones, insecticides, and beta-lactam antibiotics. The *Ch*KRED20 is a robust NADH-dependent ketoreductase identified from *Chryseobacterium* sp. *CA49*. We obtained a *Ch*KRED20 mutant (M12) through directed evolutionary screening of *Ch*KRED20, the mutant with significantly improved activity to asymmetrically reduce 4-hydroxy-2-butanone (4H2B) to (*R*)-1, 3-BDO. So far, both *Ch*KRED20 and its mutants have been expressed in intracellular in *E. coli*, the process of purification after intracellular expression is complicated, which leads to high cost. Here, we expressed M12 by constructing multicopy expression strains in *P. pastoris*, and the target protein yield was 302 mg/L in shake-flask fermentation and approximately 3.5 g/L in high-density fermentation. The recombinant M12 showed optimal enzyme activity at 30 °C and had high activity within a broad pH range of 6.0–8.0, and also showed high thermal stability. The recombinant M12 was further used for the reduction of 4H2B to (*R*)-1, 3-BDO, and 98.9% yield was achieved at 4540 mM 4H2B. The crude M12 enzyme extract was found to catalyze the bioreductive production of (*R*)-1, 3-BDO with excellent stereoselectivity (ee > 99%) and meet the production requirements. Our research shows that the M12 mutant can be used for the synthesis of (*R*)-1, 3-BDO, and the *P. pastoris* expression system is an ideal platform for the large-scale, low-cost preparation of *Ch*KRED20 or its mutants, which may have applications in industrial settings.

## 1. Introduction

As a vital C4 platform chemical, 1, 3-Butanediol (1, 3-BDO) is widely used in the field of cosmetics, acting as a solvent, and it is also a key intermediate in the synthesis of restoratives, aromas, and pheromones [1,2]. Also, 1, 3-BDO is largely demanded and can be used as an important precursor for the synthesis of butadiene, which is a chemical widely used in the manufacture of synthetic latex, resins, and rubber [3]. In addition, as a key intermediate, optical (*R*)-1, 3-BDO is very important in synthesizing aromatics, insecticides, beta-lactam antibiotics, and pheromones [3,4]. At present, the chemical approach from acetaldehyde is the main method to synthesize 1, 3-BDO; the products are racemic mixtures with *R* and *S* types [4]. (*R*)-1, 3-BDO also can be produced by microbial reduction of 4-hydroxybutanone, which is complex and costly and is not suitable for large-scale production in industry [4]. Also, (*R*)-1, 3-BDO was obtained by microbial metabolism through constructing artificial metabolic pathways in *E. coli* [5,6]. Despite the fermentative routes for the production of (*R*)-1, 3-BDO are green bioprocesses, but the titers, purification, and yields are too low for large-scale application. The production of optically pure (*R*)-1, 3-BDO by green bioprocesses using cheap and sustainable biological resources is highly desirable.

In industrial production, chemical and enzymatic methods are the main methods for preparing chiral alcohols. The enzymatic method is often favored by the industry for its advantages of simplicity of procedure, high selectivity, high conversion rate, substrate tolerance, and the eco-friendly nature of the process, compared to the chemical and synthetic biology-based methods. In the field of biocatalysis, the conversion of ketones to chiral alcohols is catalyzed by the ketoreductase (KREDs) [7] or alcohol dehydrogenase (Alcohol dehydrogenase (ADH) [8]. Some studies showed that the mutated Rhodococcus phenylacetaldehyde reductase (PAR) or Leifsonia alcohol dehydrogenase (LSADH) [9] and a short-chain carbonyl reductase (LnRCR) [10] were applied for the production of (*R*)-1, 3-BDO using 4-hydroxy-2-butanone (4H2B) as substrate.

The *Ch*KRED20 is a NADH-dependent ketoreductase identified from *Chryseobacterium* sp. *CA49*, using 2-propanol as the ultimate reducing agent [11,12]. It has been found that in the presence of the cofactor of NADH, *Ch*KRED20 or its mutants could asymmetrically reduce COBE to ethyl (*S*)-4-chloro-3-hydroxybutanoate ((*S*)-CHBE) [11] and reduce CFPO to (1*S*)-2-chloro-1-(3, 4-difluorophenyl) ethanol ((*S*)-CFPL) [7]. So far, no studies have reported that *Ch*KRED20 or its mutants could be directly used for the synthesis of (*R*)-1, 3-BDO using 4-hydroxy-2-butanone (4H2B) as substrate.

**Figure 1:**
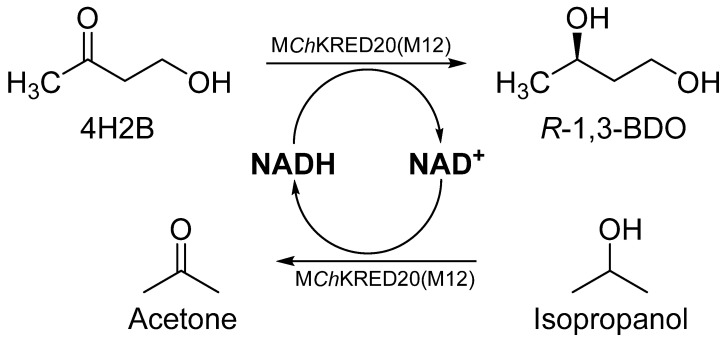
Schematic presentation of the biotransformation of 4H2B to (*R*)-1, 3-BDO. 4H2B, 4-hydroxy-2- butanone; (*R*)-1, 3-BDO, *R*-1, 3-butanediol; M*Ch*KRED20 (M12), the mutant of *Ch*KRED20.

Previous work in our lab had found that *Ch*KRED20 could serve as a catalyst that can asymmetrically reduce 4H2B to (*R*)-1, 3-BDO, but its stability and activity were poor and cannot be used as an industrial enzyme for the synthesis of (*R*)-1, 3-BDO, so we performed through directed evolutionary screening of ketoreductase *Ch*KRED20 and had obtained a mutant (M12) with significantly improved activity and thermal stability, the M12 mutant is a robust NADH-dependent ketoreductase that can asymmetrically reduce 4H2B to (*R*)-1, 3-BDO (Figure 1).

So far, recombinant expression of *Ch*KRED20 or its mutants have been expressed in intracellular in *E. coli* [11,13,14]; the process of purification after intracellular expression is complicated, which leads to high cost, and the system in bacteria cannot undergo shear modification. Due to the large market for ketoreductase, it is critical to develop low-cost methods for the large-scale purification of *Ch*KRED20 or its mutants.

For the production of heterologous proteins, such as industrial enzymes, currently, the yeast *P. pastoris* is regarded as one of the most versatile and popular expression systems [14,15]. As a secretory expression system, *P. pastoris* can simplify the production of industrial enzymes and simplify the downstream purifying process. The *P. pastoris* is suitable for high-density continuous fermentation, producing a high yield of secreted foreign proteins and with small amounts of endogenous proteins; as a single-celled microorganism, its genetic operation is easy, and it grows fast with simple nutrition [15,16]. The *P. pastoris* expression system has many advantages, such as its well-developed protein processing mechanisms, including signal peptide cleavage, protein folding, intracellular post-translational modification, and secretion of normal functional proteins into the medium [16,17,18]. In addition, production in *P. pastoris* usually uses the strong and tightly regulated AOX1 promoter, thus resulting in a heterologous protein that can account for 30% of the total cell protein when growing in methanol [18].

Previous studies showed that both *Ch*KRED20 and its mutants were expressed in *E. coli*, and no related studies have reported that *Ch*KRED20 or its mutants were expressed in *P. pastoris*. In this study, we found that the mutant of *Ch*KRED20 (M12) could asymmetrically reduce 4H2B to (*R*)-1, 3-BDO. The *Ch*KRED20 mutant M12 was successfully heterologously expressed in *Pichia pastoris*, and furthermore, multiple copy expression systems and high-density fermentation were used to improve the yield of recombinant protein to 3.5 g/L. The recombinant *Ch*KRED20 mutant M12 enzyme crude extracts were also used to produce (*R*)-1, 3-BDO, and 98.9% yield was achieved at 4540 mM 4H2B with the high optical purity of the product (ee > 99%) and met the production requirements. In this study, the *Ch*KRED20 mutant was effectively expressed and evaluated its enzymatic characteristics in *P. pastoris* for the first time and was used for the biotransformation of 4H2B to (*R*)-1, 3-BDO, which has the potential for large-scale industrial application.

## 2. Results

### 2.1. Identification of the Candidate Mutant

In our previous work, we found that *Ch*KRED20 could serve as a catalyst that can asymmetrically reduce 4H2B to (*R*)-1, 3-BDO, but its stability and activity were too poor and cannot be used as an industrial enzyme for the synthesis of (*R*)-1, 3-BDO. So, our group performed directed evolutionary screening of *Ch*KRED20, the site-saturation and combination mutation library of *Ch*KRED20 were constructed and screened, and the selection criteria were that the mutants were capable of converting of 4H2B to (*R*)-1, 3-BDO with better performance (including higher activity, tolerance to higher temperatures, tolerance to higher organic solvent concentrations) compared to the wild type. Finally, a total of 166 mutants with higher enzyme activity and thermal stability than the wild type (*Ch*KRED20) were screened, the results of the mutant activity comparison are shown in Tables S1 and S2. Among the 166 mutants, mutant NO166 (H42K, A46T, I86V, G94A, Q97K, I114V, A150Q, S153N, E173D, G185C, A187G, Y246N) showed the best activity and thermal stability, we named theNO166 mutant M12. Mutant M12 without heat treatment showed a conversion rate eight times more than that of WT, and the WT had no activity, while the M12 had a conversion rate of 96.8% when treated at 85 °C for 2 h (Table 1). The M12 mutant with significantly improved activity and thermal stability compared to the wild type. Therefore, in this study, we took M12 as the research object to further study its expression in *P. pastoris* and its enzymatic characteristics.

The steady-state kinetic parameters of the WT and mutant M12 were determined at 40 °C. The Michaelis–Menten constants (Km), catalytic constants (kcat), and catalytic efficiencies ratios (kcat/Km) of WT and M12 using 4H2B as the substrate were summarized in Table 1. The results showed that the mutant M12 had a higher catalytic frequency (kcat) of 387.33 s^−1^, over a four-fold improvement from the wild type. The Michaelis–Menten constant (Km) of the mutant M12 decreased to 35% of the WT, indicating better affinity with the substrate, which further increased the catalytic efficiency (kcat/Km) of the mutant M12 to 13.7-fold of that of the WT (Table 2).

### 2.2. Molecular Docking and Active Cavity Analysis of the Mutant ChKRED20 (M12)

In our previous work, we found that *Ch*KRED20 could serve as a catalyst that can asymmetrically reduce 4H2B to (*R*)-1, 3-BDO, but its stability and activity were too poor and cannot be used as an industrial enzyme for the synthesis of (*R*)-1, 3-BDO. Therefore, we performed directed evolutionary screening of *Ch*KRED20 and obtained an M12 mutant *(*H42K, A46T, I86V, G94A, Q97K, I114V, A150Q, S153N, E173D, G185C, A187G, Y246N) with significantly improved activity and thermal stability. To analyze the reasons why the enzyme activity of the M12 mutant was higher than that of the wild type (WT)*,* molecular docking was performed with the substrate of 4H2B and NAD^+^. 

**Figure 2:**
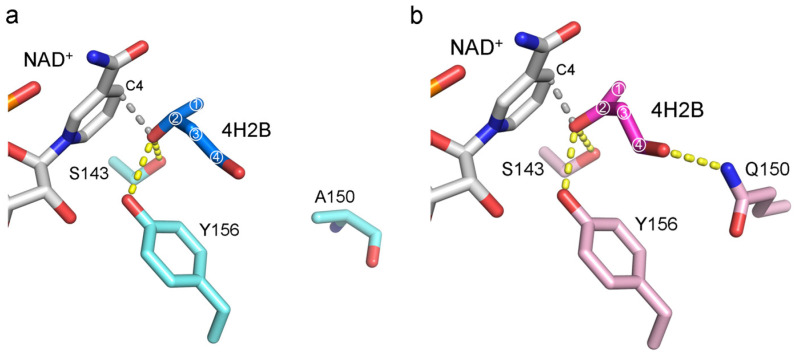
The LigPlot+ analysis results of protein-ligand (4H2B) interactions for the wild-type *Ch*KRED20 (**a**) and the mutant M12 (**b**). The four carbon atoms of 4H2B were represented as ①, ②, ③, and ④ in the figure. The C4 atom of the nicotinamide, where the hydride transfer occurs, makes a close approach to 4H2B, forming van der Waals interactions with 4H2B. The dashed gray lines in the figure represent van der Waals interactions, the yellow dashed lines indicate the hydrogen bonds.

**Figure 3:**
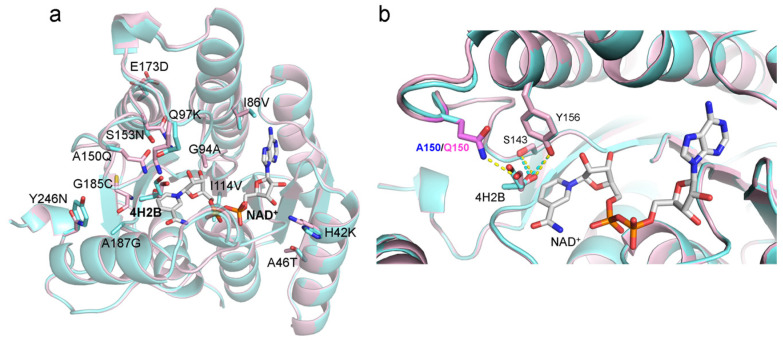
Overlaying the structures of molecule-docked complexes to compare the interactions between the wild-type (cyan) and mutant M12 (light pink) proteins and the substrate 4H2B. (**a**) The 12 mutation sites in the proteins are depicted as sticks in the diagram. (**b**) The amino acid residue of *Ch*KRED20 (WT in cyan/Mut in light pink) that interacts with the substrate 4H2B is shown in stick form. The coenzyme NAD^+^ and the substrate are also represented in stick form, with hydrogen bonds indicated by dashed lines: cyan for WT and yellow for Mut.

The CB-dock2 software (https://cadd.labshare.cn/cb-dock2/ accessed on 13 September 2024) [19] was used to dock the substrate 4H2B into the active site of the protein *Ch*KRED20 in the presence of the coenzyme NAD^+^ to reveal key information such as binding patterns and binding strength, etc. The interactions between the substrate and the protein were analyzed using the LigPlot^+^ v.2.2 software (http://www.ebi.ac.uk/thornton-srv/software/LigPlus accessed on 13 September 2024) [20,21], as illustrated in Figure 2. The complex structures in Figure 3 depict the 12 mutation sites of the mutant protein. These include residues H42K, I86V, G94A, and I114V, situated near NAD^+^, as well as Q97K, A150Q, S153N, and A187G located at the substrate binding site.

A comparative analysis of molecular docking results showed that the substrate positions in wild-type and mutant protein complexes were similar. In both complexes, the substrate was adjacent to the coenzyme NAD^+^, forming hydrophobic interactions. The orientation of the substrate’s carbonyl group towards the NAD^+^ nicotinamide ring facilitates reduction reactions. Additionally, in both protein types, the substrate’s carbonyl group forms stabilizing hydrogen bonds with the hydroxyl groups of Y156 and S143 within the protein-NAD complex; the C4 atom of the nicotinamide, where the hydride transfer occurs, makes a close approach to 4H2B, forming van der Waals interactions with 4H2B (Figure 2).

A notable difference between the wild-type and mutant proteins lies in residue Q150. The elongated polar side chain of Q150 in the mutant protein allows additional hydrogen bonds with the substrate’s 4-hydroxyl group, enhancing substrate stabilization. In contrast, the methyl side chain of A150 in the wild-type protein is further from the substrate (Figure 2), leading to weaker binding interactions. As a result, the mutant protein shows significantly higher substrate affinity compared to the wild-type protein.

Binding energy calculations using YASARA [22] indicated that the wild-type protein has a binding energy of 30.37 kJ/mol, while the mutant has a binding energy of 40.25 kJ/mol. The energy difference of approximately 10 kJ/mol, close to the average energy of an N-H…O hydrogen bond (around 8 kJ/mol), is primarily due to the hydrogen bond between the substrate’s 4-hydroxyl group and the N–H of the Q150 side chain.

These findings correlated with our analysis of substrate-protein interactions and were consistent with the difference in enzymatic activity between the WT and mutant, demonstrating that mutant proteins enhance enzymatic activity through improved substrate binding.

### 2.3. Muti-Copy Expression of M12 Using P. pastoris GS115

**Figure 4:**
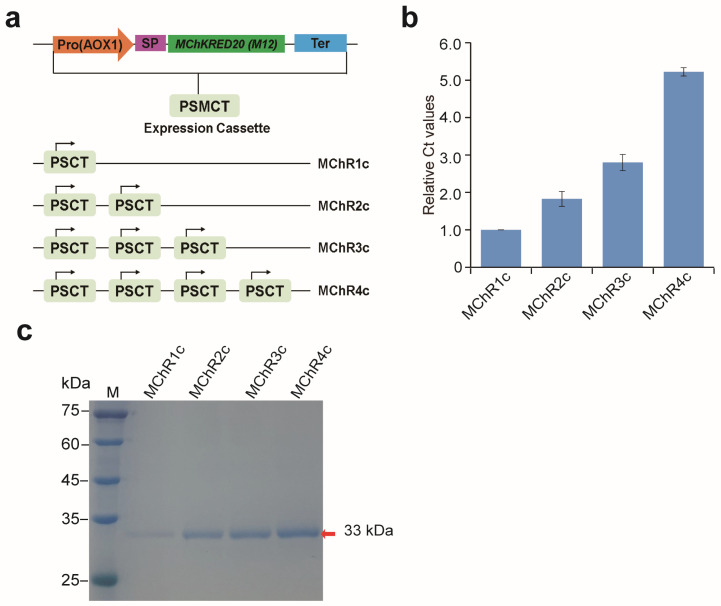
Muti-copy expression of M*Ch*KRED20 (M12) using gene dosage. (**a**) Strategy of multi-copy strain construction. (**b**) The qPCR of the M12 expression cassettes integrated into the host strains carrying 1–4 copies. (**c**) SDS-PAGE analysis of M12 secreted into the culture supernatant. M: protein marker; lanes 1–4: supernatants from shake-flask fermentation of MChR1c, MChR2c, MChR3c, and MChR4c strains, respectively, collected after 144 h induction with 1% methanol.

The ketoreductase M12 contains 249 amino acids and encodes a protein of approximately 27 kDa. To improve the protein expression level of the recombinant M12, multi-copy expression vectors carrying one to four copies of the M12 expression module were constructed using the biobrick assembly method (Figure 4a). These vectors were linearized and transformed into *P. pastoris* GS115 and selected by histidine-free MD plates. The positive recombinant *P. pastoris* strains were named MChR1c, MChR2c, MChR3c, and MChR4c (single-copy, 2-copy, 3-copy, and 4-copy, respectively).

To confirm the target gene copy number in recombinant strains, qPCR was carried out. The relative signal of *MKRED*/*GAP* to MChR2c, MChR3c, and MChR4c was 2.02, 2.97, and 5.22 times higher than that of MChR1c (Figure 4b), thus suggesting that *MKRED* was present in multiple copies in the MChR2c, MChR3c, and MChR4c strains. 

Then, MChR2c, MChR3c, and MChR4c strains were cultured using shake-flask fermentation, and the expression levels of M12 in the multi-copy expression strains were analyzed with 12% SDS-PAGE. After 144 h methanol induction, the main band of all samples was approximately 33 kDa, and the band intensities enhanced with the increase of *MKRED* gene copy number, and the MChR4c strain had the highest protein expression level (Figure 4c). These results indicated that the yield of recombinant M12 was positively correlated with the copy number of the *MKRED* gene in *P. pastoris*.

### 2.4. Shake-Flask and High-Density Fermentation Using the MChR4c Strain

**Figure 5:**
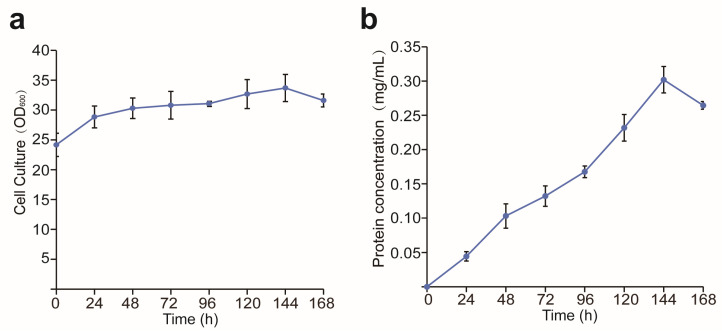
Quantitative analysis of the expression of M12 produced by shake-flask fermentation. Time course of cell density (**a**) and the yield of the target protein (**b**) during the induction phase.

**Figure 6:**
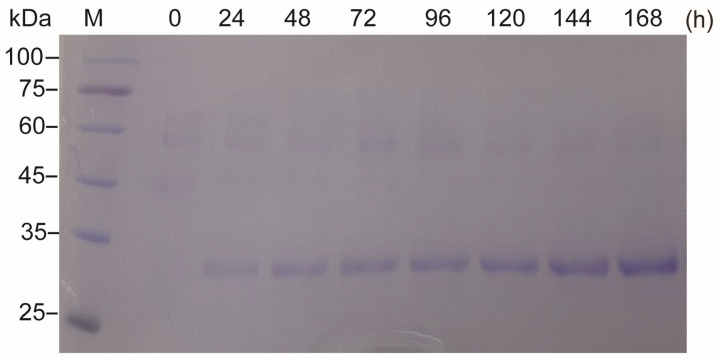
SDS-PAGE analysis of M12 secreted into the supernatant during high-density fermentation. M: protein molecular weight marker (the molecular weight of each band is indicated on the left); cell culture supernatant after induction with 1% methanol for 0, 24, 48, 72, 96, 120,144, and 168 h, respectively, all samples were diluted 10 times.

As the MChR4c strain had the highest protein expression level, the MChR4c strain was selected for subsequent experiments. During shake-flask fermentation, at the beginning of the induction, the OD_600_ of the cell culture was about 24 and reached a maximum of approximately 33 after 144 h (Figure 5a). The concentration of recombinant protein was related to cell growth and reached 302 mg/L after being induced for 144 h (Figure 5b). In the high-density fermentation process, after 168 h, the yield of the target protein reached a maximum of about 3.5 g/L (Figure 6); compared with shaker fermentation (Figure 5b), high-density fermentation using a fermenter increased the expression of the target protein by more than 10 times (Figure 5b). Because *P. pastoris* can be used for high-density fermentation, the expression of the target protein is also affected by the fermentation; the expression level of foreign proteins may increase significantly, and some proteins can even be increased by 20 times or more compared with the fermentation in shaking bottle [18,23].

### 2.5. The Glycosylation Modification of Recombinant M12

**Figure 7:**
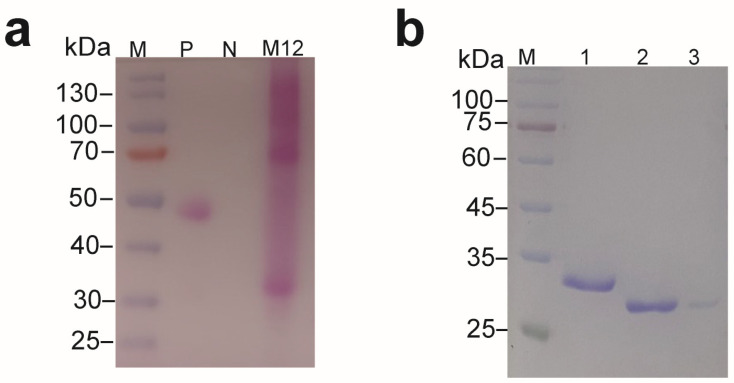
The glycosylation staining and deglycosylation of M12. (**a**) Glycoprotein staining to analyze the expression of M12 expressed in *Pichia pastoris*, P: the positive control, N: the negative control, M12: the recombinant M12 (**b**) SDS-PAGE to analyze the M12 expressed in *Pichia pastoris* after treated with Endo H, lane 1: M12, lane 2: M12 after treated with Endo H, lane 3: the Endo H.

The recombinant M12 protein was purified and concentrated to investigate the enzyme characteristics, and the molecular weight of the recombinant M12 was significantly larger than predicted. Glycosylation is the most common post-translational modification of proteins secreting in *P. pastoris*, which leads to increasing the molecular weight of the protein [12,16]. Therefore, we detected the glycosylation modification with a Glycoprotein Staining Kit. Glycosylation staining showed that the target protein was severely glycosylated (Figure 7a), and the molecular weight of recombinant M12 was decreased to approximately 27 kDa after being treated with Endo H, which was consistent with what was predicted. After treatment with Endo H, the molecular weight decreased to the same size as the enzyme expressed in *E. coli* (Figure 7b). These results suggested that the M12 protein expressed in *P. pastoris* was modified by glycosylation.

### 2.6. The Characteristics of Recombinant MChKRED20

**Figure 8:**
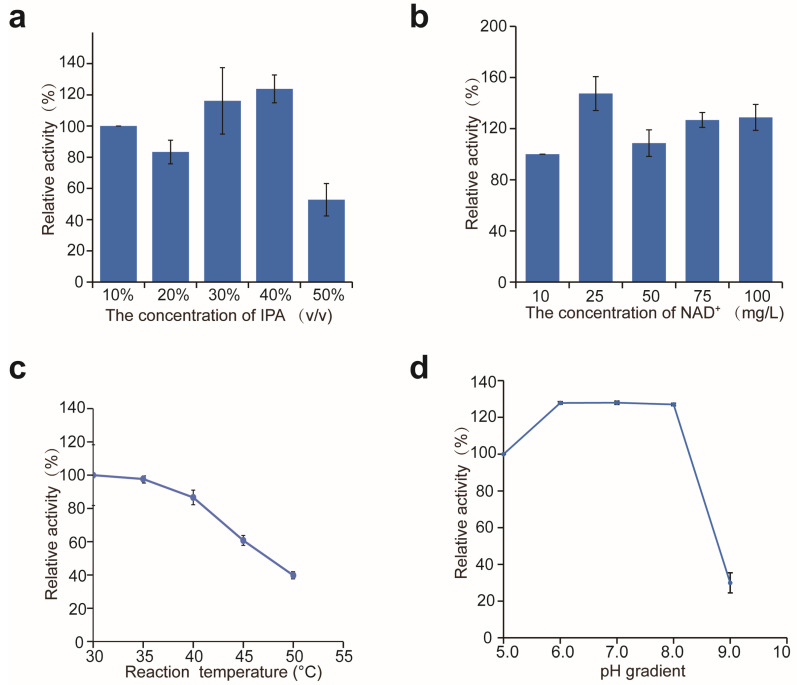
The optimal concentration of IPA (**a**) and NAD^+^ (**b**) in the reaction system. (**c**) Temperature optimization for M12 activity. Enzyme activity at 30 °C was set to 100%. (**d**) pH optimization for M12 activity. Enzyme activity at pH 5.0 was set to 100%.

Studies have shown that wild-type *Ch*KRED20 has strong antioxidant activity against isopropyl alcohol, which can be used as both solvent and co-substrate for recovery cofactors; when biotransformating COBE to (*S*)-CHBE, it could be used in the reaction system up to 75% (*v*/*v*) [13]. To find the optimal concentration of isopropanol (IPA) in the reaction system for the *Ch*KRED20 mutant M12 to biotransformation of 4H2B to (*R*)-1, 3-BDO, we carried out the biotransformation reaction using 10–50% (*v*/*v*) of isopropanol in the reaction system, results showed that up to 40% (*v*/*v*) of isopropanol could be used in the reaction system (Figure 8a), which facilitated the application of the biphasic system. This suggests that the optimal concentration of IPA may be different for different reaction substrates. Furthermore, the optimum concentration of NAD^+^ in the reaction system was measured; when the NAD^+^ was 25 mg/L, the recombinant M12 showed the highest activity (Figure 8b). 

**Figure 9:**
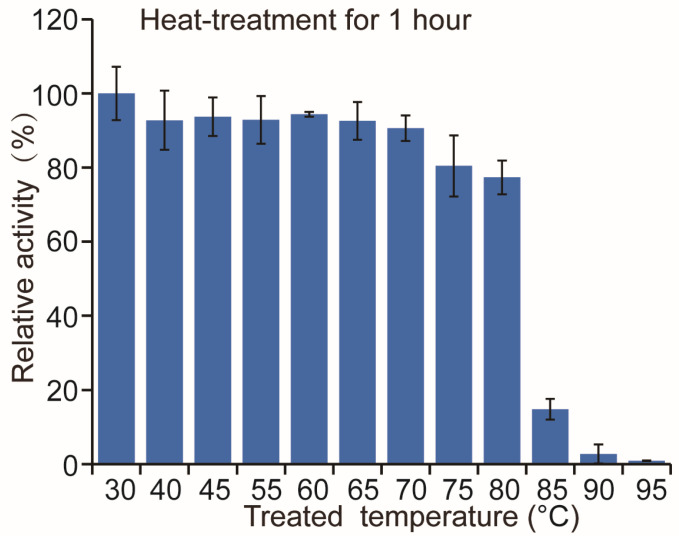
Thermostability of the recombinant M12. The initial enzyme activity was set to 100%.

The enzyme activity of M12 was performed at 30–50 °C and a pH range of 5.0–9.0. The M12 showed maximum activity at 30 °C, and when the temperature was above 50 °C, the activity decreased dramatically (Figure 8c). The M12 had high activity within a broad pH range of 6.0–8.0 (Figure 8d). The M12 also had relatively high thermostability; after incubation at 75 °C for 1 h, the enzyme activity remained above 80% (Figure 9). It has been reported that glycosylation modification can improve the thermal stability of enzymes [24,25], so the glycosylation modification of M12 in *P. pastoris* may enhance the thermostability of the protein.

### 2.7. Biotransformation of 4H2B to (R)-1, 3-BDO Using M12

**Figure 10:**
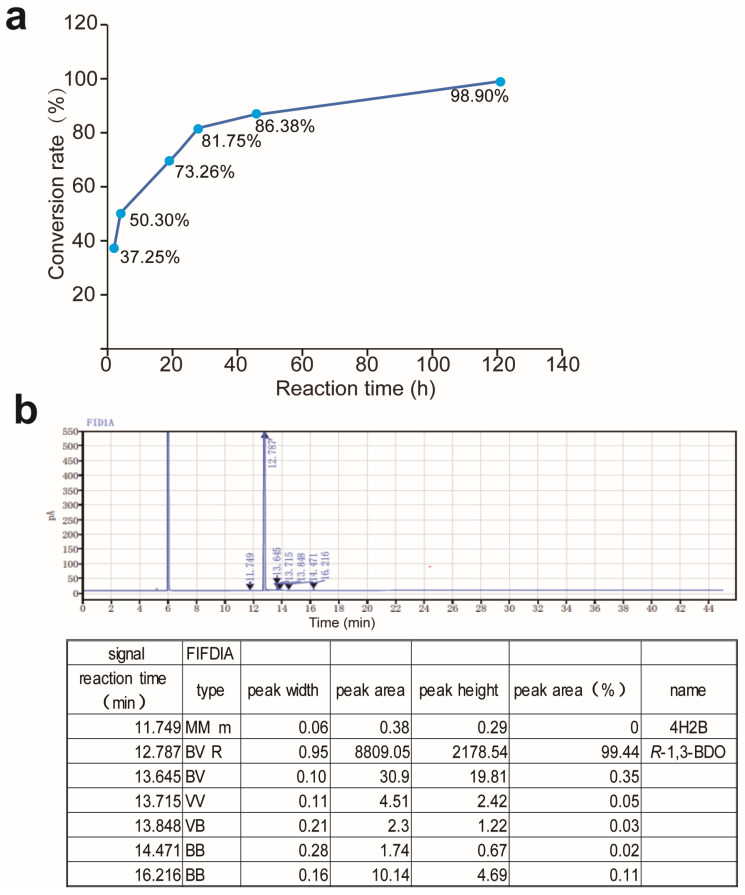
Biotransformation of 4H2B to (*R*)-1, 3-BDO. (**a**) The conversion rate over the time using the crude enzyme extract of M12. (**b**) The purity of the production of (*R*)-1, 3-BDO analyzed with GC.

Based on the characteristics of the M12, the biotransformation of 4H2B to (*R*)-1, 3-BDO was carried out at an elevated temperature of 40 °C. A 100 mL reduction reaction with 400 g/L 4H2B (4540 mM) was performed. The time courses of the biotransformation of 4H2B were measured in a two-phase system containing 40% (*v*/*v*) isopropyl alcohol using 3 g/L of crude enzyme extracts. The reaction proceeded smoothly almost no by-products were produced, and the transformation was complete after 120 h. The conversion rate achieved 98.9% (Figure 10a). The product (*R*)-1, 3-BDO was obtained with excellent enantioselectivity (ee > 99%) (Figure 10b).

## 3. Discussion

The *Ch*KRED20 or its mutants were used for the enzymatic synthesis of chiral alcohols, such as (*R*)-3, 5-bis(trifluoromethyl)-1-phenylethanol [10] and ethyl (*S*)-4-chloro-3-hydroxybutanoate [13], 1, 3-BDO is an important C4 diol that is widely used as a solvent in cosmetics and as a monomer in the polymer industry. At present, three enzymatic methods were reported to be used for the preparation of (*R*)-1, 3-BDO [10], such as the dehydrogenase was used to oxidize the (*S*)-1, 3-BDO enantiomer in racemate to (*R*)-1, 3-BDO [26], or the enantio-selective reduction of 4H2B to (*R*)-1, 3-BDO [27], the short-chain carbonyl reductase (LnRCR) mutant was used for the reduction step to produce (*R*)-1, 3-BDO [10]. 

So far, no studies have reported that *Ch*KRED20 or its mutants can be used in the synthesis of (*R*)-1, 3-BDO. Previous work in our lab had found that *Ch*KRED20 could serve as a catalyst that can asymmetrically reduce 4H2B to (*R*)-1, 3-BDO, but its stability and activity were poor and cannot be used as an industrial enzyme for the synthesis of (*R*)-1, 3-BDO, so we obtained a mutant M12 with significantly improved activity and thermal stability through directed evolution screening. However, the mechanism by which M12 mutant activity was higher than wild-type *Ch*KRED20 is not clear. In this study, we performed the molecular docking of *Ch*KRED20 and the M12 mutant with the 4H2B substrate. The M12 mutant protein shows significantly higher substrate affinity compared to the wild-type protein, and the mutant had a higher binding energy of 40.25 kJ/mol than that of the wild-type protein (30.37 kJ/mol). The energy difference of approximately 10 kJ/mol, close to the average energy of an N–H…O hydrogen bond (around 8 kJ/mol), was primarily due to the hydrogen bond between the substrate’s 4-hydroxyl group and the N-H of the Q150 side chain. Further, we performed the steady-state kinetic parameters of the wild-type *Ch*KRED20 and mutant M12, and the results indicated that the M12 had a better affinity with the substrate, which further increased the catalytic efficiency. The mutant not only improves the enzyme binding to the substrate but also enhances the enzyme catalytic efficiency. In the future, we can further screen mutants with tighter binding and higher affinity to 4H2B to obtain more mutants with better enzymatic activity for the synthesis of (*R*)-1, 3-BDO. Our study may also provide a way to screen and optimize other enzymes for the high-efficiency mutants.

With the study of the regulation of metabolic capacity [28], safety, and function (*R*)-1, 3-BDO is increasingly used in the food and health products industry. Our study found that the *Ch*KRED20 mutant M12 was a key enzyme that could be used to produce (*R*)-1, 3-BDO from 4H2B in industrial production, and its expression host safety is particularly important. Previously, *Ch*KRED20 or its mutants were reported to be expressed in intracellular in *E. coli*, leading to cumbersome purification steps and high purification costs in the large-scale preparation. Endotoxin was produced in *E. coli*, while *P. pastoris* is generally considered to be a safe (GRAS) strain with extraordinary protein-secreting capabilities [18,29]. The *P. pastoris* has been successfully used as a host for the secretory expression of heterologous proteins, and accordingly, it may be suitable for the production of *Ch*KRED20 or its mutants in yeast rather than in bacteria. In this study, we achieved high-level expression of the *Ch*KRED20 mutant using the *P. pastoris* system and assessed its reduction activity to produce (*R*)-1, 3-BDO.

As an industrial enzyme, it is necessary to reduce its production cost and increasing protein expression is one way to reduce production costs. In addition to codon optimization and the use of a strong AOX1 promoter, the copy number of the heterologous gene expression cassette is also an important way to achieve efficient protein expression [18,23,29]. In this study, multi-copy expression was successful at obviously improving the yield of recombinant *Ch*KRED20 mutant M12. The purification cost is also an important factor to be considered in industrial enzyme production. So far, *Ch*KRED20 or its mutants were intracellularly expressed in *E. coli*, and it needed to break the cell wall and heat treatment to obtain target proteins, which would increase the cost of production, and heat treatment affects the enzyme activity. Large amounts of intrinsic proteins are not secreted in *P. pastoris*, while foreign protein is secreted to the medium, which can account for more than 90% of the total in the medium, so it is very easy to isolate the target protein [16]. Therefore, the crude enzyme can be used directly without purification and simple desalination treatment, which can reduce the cost of production. Moreover, glycosylation modification in *P. pastoris* can improve the thermal stability of the enzyme [25].

In conclusion, we have successfully expressed the *Ch*KRED20 mutant M12 protein in *P. pastoris* and improved the expression level of the target protein by constructing muti-copy strains carrying four copies of the target gene. The protein yield was 302 mg/L by shaking flask fermentation and 3.5 g/L by high-density fermentation. The recombinant *Ch*KRED20 mutant M12 showed high thermostability, and the crude enzyme extract was efficient in the bioreductive production of (*R*)-1, 3-BDO with a high conversion rate (98.9%) and stereoselectivity (ee > 99%). Zheng et al. reported that they used a *C. krusei* cell catalyst to convert 45 g/L (510 mM) of 4H2B to (R)-1, 3-BDO with 99% ee, achieving a yield of 83.9% in about 50 h [27]. Recombinant dehydrogenase was also reported to be used for the transformation of 4H2B to (*R)*-1,3-BDO with the 250 g/L (2837 mM) of 4H2B, and the (*R)*-1, 3-BDO product yield was 99% with 99% ee when the transformation time was 500 h [9]. Our results suggested that the recombinant M12 expressed in *P. pastoris* had the highest efficiency and conversion rate of (*R*)-1, 3-BDO production when using 4H2B as substrate compared to other enzymatic conversion methods reported so far.

Studies found that *Ch*KRED20 or its mutants were responsible for the enzymatic synthesis of (*R*)-3, 5-bis(trifluoromethyl)-1-phenylethanol [10] and ethyl (*S*)-4-chloro-3-hydroxybutanoate [13], which are key intermediates for the chiral drug. In our study, an efficient and functional expression system for the production of *Ch*KRED20 mutant will not only facilitate further studies of *Ch*KRED20 or its mutants but also pave the way for possible large-scale production of ketoreductases in the near future and provide a possibility for the enzymatic synthesis of more chiral alcohols. The X-ray crystal structure of *Ch*KRED20 was refined, and mutants were designed based on structure, which increased the diversity of enzyme reaction substrates [11], and some mutants of *Ch*KRED20 have been obtained to improve some of its properties, including improved thermal stability [13]. Therefore, our study provides an idea for the expression and application of these *Ch*KRED20 mutants. In the future, more work on recombinant *Ch*KRED20 mutants with higher purity and more activity should be studied, and improve the efficiency of (*R*) 1, 3-BDO with ketoreductases to further decrease the production cost.

## 4. Materials and Methods

### 4.1. Strains, Reagents and Media

*E. coli* XL10-Gold, BL21(DE3), and *P. pastoris GS115* strains were purchased from Invitrogen (Carlsbad, CA, USA). The pET-23a (+) used as the expression vector was maintained in our laboratory. The expression vector, pHBM905BDM plasmid [23], was constructed and stored in our laboratory. The *P. pastoris* culture media, including minimal dextrose (MD), buffered glycerol-complex (BMGY), and buffered methanol-complex (BMMY), were prepared as described in the *P. pastoris* expression manual (Invitrogen, Carlsbad, CA, USA) [18,23,30]. 

### 4.2. The Construction of Mutant Library

The Quikchange kit (Agilent, Santa Clara, CA, USA) was used. The sequence design of the mutagenesis primers was performed according to the instructions of the kit. The construction of the site-saturation mutagenesis library was as follows. The PCR reaction consisted of 10 µL of 5× buffer, 1 µL of 10 mM dNTP, 1 µL of plasmid DNA template (50 ng/µL), 0.75 µL (10 µM) each of the upstream and downstream primers, 0.5 µL Phusion and 36 µL of ddH_2_O, the PCR primer has an NNK codon at the mutation position. The PCR amplification was performed as follows: 98 °C for 3 min and then 25 cycles of 98 °C for 10 s (denaturation), 72 °C for 3 min (annealing and extension), 72 °C for 7 min (extension), cooling to 4 °C. Then the PCR product was digestion at 37 °C for 4 h by adding 2 µL of DpnI to eliminate the plasmid template. The digested PCR product was then transformed into *E. coli* BL21(DE3) competent cells and plated on LB agar plates with chloramphenicol to obtain a site-saturation mutagenesis library.

### 4.3. High-Throughput Screening of Ketoreductase ChKRED20 Mutant Library

The expression of the ketoreductase mutant library was performed in 96-well plates. The operation procedure was as follows: mutant colonies were picked and inoculated into LB medium with chloramphenicol in a 96-well shallow plate (150 µL per well), then cultured for 12–18 h at 180 rpm, 30 °C. When OD_600_ of shallow plate culture reached 2.0, 20 µL of this culture was transferred to a TB medium (containing 400 µL of TB medium and 6 g/L of lactose per well) in a 96-well deep plate as expression culture, and it was shaken overnight for 18 to 20 h at 240 rpm under 30 °C. The expression culture was centrifuged (4000 rpm, 10 min) to collect cell pellets. Next, 200 µL/well of cell lysis buffer (100 mM phosphate buffer, pH 7.5, containing 1 mg/mL lysozyme) was added to the deep well plate, and the plate was sealed, placed on a plate shaker at 700 rpm for 1 h to break the cells. Then, the cell lysate was centrifuged (4000 rpm, 10 min), and 160 µL/well of supernatant enzyme solution was collected into a new plate, which was subsequently sealed and subjected to heat treatment by shaking in a water bath shaker at 72 °C for 2.5 h.

The enzyme solution was centrifuged (4000 rpm,15 min) after the heat treatment, then the supernatant (30 µL) was transferred to a 96-well plate, which was pre-loaded with reaction stock solution (170 µL/well). The reaction plate was then heat-sealed with an aluminum film and placed in a shaker at 45 °C, 200 rpm to start the reaction. After 15 h of reaction, 50 µL of the reaction solution was transferred into a new 96-deep well plate, and the reaction was quenched by adding 1 mL of ethyl acetate. The reaction was shaken on a plate shaker for 30 min (800 rpm), then centrifuged (4000 rpm, 30 min), and the supernatant was subject to GC analysis to determine the conversion.

### 4.4. Measurement Enzyme kineticsPurified Enzymes Were Used

All measurements were performed in triplicate. To determine the kinetic parameters of the enzyme for 4H2B, WT (0.5 μM)/M12 (0.1 μM) was pre-incubated with 4H2B to a concentration of 1 mM–750 mM in the 100 mM potassium phosphate buffer (pH 7.0) for 90 s at 40 °C. The reaction was then initiated by adding NADH (0.25 mM), bringing the final volume of the assay to 200 µL, and monitored by measuring the decrease in absorbance at 340 nm using a spectrophotometer. Data were fitted to the Michaelis–Menten equation using OriginPro 2021 to generate the estimates of Km, Vmax, and kcat.

### 4.5. Molecular Docking

The three-dimensional structures of both the wild type (*Ch*KRED20) and mutant (M12) were generated using the AlphaFold Server (Google DeepMind). Upon providing the peptide sequence, structure prediction was performed under the conditions specifying four protein copies (referencing the homologous structure assembly of 6IXM) and designating the substrate NAD^+^ as the ligand. The CB-dock2 software (https://cadd.labshare.cn/cb-dock2/ accessed on 13 September 2024) [19] was used to dock the substrate into the active site of the protein *Ch*KRED20. The interactions between the substrate and the protein were analyzed using the LigPlot^+^ software [20,21]. Furthermore, the binding energy between the substrate and the protein was calculated using YASARA [22]. All of the protein structures were visualized and analyzed with PyMOL(TM) 3.0.2 and Discovery studio 2021.

### 4.6. Construction of pHBM905BDM-MKRED Expression Vectors

The mutant of *Ch*KRED20 (M12) (gene, *MKRED*) was codon optimized based on the *P. pastoris* system and was synthesized by Sangon BioTech (Shanghai, China). The *MKRED* gene was cloned into the pHBM905BDM vector at the *Cpo* I–*Not* I site to generate plasmid pHBM905BDM-MKRED1 with the TLTC DNA cloning method [31]. The multi-copy expression vectors of M*Ch*KRED20 were constructed by the biobrick assembly method [30]. The recombinant vectors were verified by sequencing, named pHBM905BDM-MKRED2, pHBM905BDM-MKRED3 and pHBM905BDM-MKRED4.

### 4.7. Screening of Recombinant Yeast Strains Expressing M12

The recombinant plasmids were linearized with *Sal* I, purified, and then transformed into competent cells of *P. pastoris* GS115 through electroporation. The positive transformants were selected on MD plates and further identified by colony PCR with specific primers to amplify the fragment of the *MKRED* gene. 

### 4.8. Determination of MKRED Gene Copy Numbers

The genomic DNA of yeast was extracted with a Yeast Genomic DNA Isolation Kit (Omega, Rhinebeck, NY, USA). Verification of the gene copy number was carried out as previously described [30]. The glyceraldehydes-3-phosphate dehydrogenase (*GAP*) gene of *P. pastoris* was used as an internal reference gene for qPCR. Using *GAP* as an internal reference gene, the copy number of *MKRED* was determined by the 2^−ΔΔCt^ method [30]. 

### 4.9. Expression of the Recombinant M12 Using Shake-Flask Fermentation

Recombinant *P. pastoris* strains containing different copies of the target gene were cultured in shaking flasks of 100 mL BMGY medium for 48 h at 28 °C; cells were harvested after centrifugation at 4 °C for 5 min (4000× *g*), then inoculated in 50 mL BMMY medium, and methanol with a final concentration of 1% (*v*/*v*) was added every 24 h to induce foreign protein expression. Fermentation was terminated after induction for 144–168 h; supernatants were collected and centrifuged at 4 °C for 5 min (10,000× *g*). The different samples were prepared and then detected with 12% (*w*/*v*) sodium dodecyl-sulfate polyacrylamide gel electrophoresis (SDS-PAGE) and staining with Coomassie Brilliant Blue G-250. The protein concentrations were determined by the Bradford kit (Beyotime, Shanghai, China) [23]. 

### 4.10. High-Density Fermentation of Recombinant Strain

Fed-batch fermentation was carried out according to the Invitrogen Pichia Fermentation Process Guidelines and previously described methods [18,30]. The recombinant strain was cultured in 200 mL YPD medium as the seed medium at 28 °C for 24 h. Then, the seed medium was transferred to a 5 L fermenter containing 2 L of BSM medium. The fermentation was performed at 28 °C, pH 5.8, and 25–30% dissolved oxygen (DO) in the early stages. DO was rapidly increased to 100% when glycerol was exhausted, then 50% (*v*/*v*) glycerol containing PTM trace salts (12 mL/L) was added at a rate of 12 mL/h/L to continue cell growth. When the OD_600_ reached about 300, 1% methanol with PTM trace salts (12 mL/L) was fed at a rate of 3 mL/h/L to induce the expression of foreign protein. In the induction phase of fermentation, the conditions were adjusted to 25 °C and pH 5.0, and 30–35% DO was maintained. Approximately 30 mL cultures were collected per 12 h for protein concentration detection assay until the end of fermentation [18,23].

### 4.11. Glycoprotein Staining and Deglycosylation of the Recombinant Proteins

To perform glycoprotein staining, different samples were treated and separated via SDS-PAGE by 12% (*w*/*v*) polyacrylamide gels, then glycoprotein staining was carried out following the method described in the Glycoprotein Staining Kit (Beyotime, Shanghai, China). 

Deglycosylation of the glycoproteins was performed as previously described [32,33]. Briefly, the fermentation supernatant was collected after 144 h induction with methanol. The target glycoproteins and recombinant Endo H were mixed in a 10:1 concentration ratio and incubated at 37 °C for 2 h, and then the samples were detected by 12% SDS-PAGE.

### 4.12. Enzymatic Characterization of M12

All experiments were performed in triplicate. The enzyme activity of M12 was measured using 4-hydroxy-2-butanone(4H2B) as the substrate. Purified enzymes were used to determine the enzyme activity. Asymmetric reduction of 4H2B was performed at 40 °C in a two-phase system in which 60% (*v*/*v*) potassium phosphate buffer (100 mM, pH 7.0) was dissolved with 0.025 g NAD^+^/L and 3 g/L purified enzyme, 40% (*v*/*v*) of the isopropyl alcohol phase was dissolved with 100 g 4H2B/L (1135 mM) for a total volume of 0.2 mL. The products were extracted with ethyl acetate after reaction for 6 h with shaking at 400 rpm and analyzed with Gas chromatography (GC).

To measure the optimal concentration of the isopropyl alcohol (IPA)in the reaction system, the reaction was performed at a range of IPA concentrations (10% (*v*/*v*)–50% (*v*/*v*)), and the reaction was performed at 40 °C. To determine the optimal concentration of NAD^+^ in the reaction, different concentrations of NAD^+^ (10–100 mg/L) were added to the reaction system, and the reaction was performed at 40 °C. 

To measure the optimal temperature of the M12 enzyme, the reaction was performed at a range of temperatures (30–50 °C). To determine the optimal pH of the M12 enzyme, the reaction was performed at 40 °C, as pH 5.0 and pH 9.0 are far from the p*K*a of the phosphate buffer and near the equivalence point [34], so phosphate buffer may not be suitable for maintaining a constant pH at pH 5.0 and pH 9.0; therefore, different pH buffers were used to regulate the reaction system with sodium acetate (100 mM, pH 5.0–6.0), phosphate buffer (100 mM, pH 7.0) and Tris-HCl (100 mM, pH 8.0–9.0).

To analyze the thermostability of M12, the purified enzymes were incubated at different temperatures for 1 h, then followed by immediate chilling on ice for 5 min and measured at 40 °C as the method described above; the remaining enzyme activity was measured to indicate thermal stability.

### 4.13. Bioreduction of 4H2B to (R)-1, 3-BDO Using Crude Enzyme Extracts

Asymmetric reduction of 4H2B to (*R*)-1, 3-BDO were performed at 40 °C in a two-phase system a total volume of 100 mL, the biphasic system containing 60% (*v*/*v*) of the phosphate buffer (100 mM, pH 7.0) dissolving 0.025 g NAD^+^/L and 3 g/L of the crude enzyme extracts, and 40% (*v*/*v*) of the isopropyl alcohol phase dissolved with 400 g/L 4H2B (4540 mM). The reaction was traced over 120 h, and the conversion rate was calculated by detecting the content of the substrate remaining after different reaction times; the products were extracted with ethyl acetate and analyzed with Gas chromatography (GC) to test their purity.

## 5. Conclusions

In this study, we found that the mutant of *Ch*KRED20 (M12) could asymmetrically reduce 4H2B to (*R*)-1, 3-BDO. Furthermore, we performed the molecular docking of *Ch*KRED20 and the M12 mutant with the 4H2B substrate and found that the mutant M12 protein showed significantly higher substrate affinity and tighter binding compared to the wild-type protein, the molecular docking results explained the mechanism of why *Ch*KRED20 mutant M12 showed higher enzyme activity in the synthesis of (*R*)-1, 3-BDO from 4H2B than wild-type. Our study may also provide a way to screen and optimize other enzymes for the high-efficiency mutants.

Previous studies showed that both *Ch*KRED20 and its mutants were expressed in *E. coli*, and no related studies have reported that *Ch*KRED20 or its mutants were expressed in *P. pastoris*. In this study, the *Ch*KRED20 mutant M12 was successfully heterologous expressed in *Pichia pastoris*, and meanwhile, a multiple copy expression system and high-density fermentation were used to improve the yield of recombinant protein to 3.5 g/L. The recombinant *Ch*KRED20 mutant enzyme crude extracts were also used to produce (*R*)-1, 3-BDO, and 98.9% yield was achieved at 4540 mM 4H2B with the high optical purity of the product (ee > 99%) and meet the production requirements. In this study, the *Ch*KRED20 mutant was effectively expressed and evaluated its enzymatic characteristics in *P. pastoris* for the first time and was used for the biotransformation of 4H2B to (*R*)-1, 3-BDO, which has the potential for large-scale industrial application.

## 6. Patents

Enzymaster (Ningbo) Bio-engineering Co., Ltd. has applied for a patent (application no. 202410427822.1) for high expression of keto reductase in *P. pastoris* and its application and W.C., B.S., H.C., X.W., L.S., L.Z., C.H., L.Z., Q.P., S.L., and C.Z. listed as co-inventors.

## Figures and Tables

**Table 1:** Comparison of enzyme activity and thermal stability between the mutant M12 and wild type (WT).

Enzyme	Enzyme Concentration	Conversion Rate	Product (ee)
WT (without heat treatment)	30% *v*/*v*	44.9%	>99%
WT (treated at 85 °C for 2 h)	30% *v*/*v*	0	--
M12 (without heat treatment)	8% *v*/*v*	99.1%	>99%
M12 (treated at 85 °C for 2 h)	8% *v*/*v*	96.8%	>99%

**Table 2:** Steady-State kinetic parameters of the wild-type *Ch*KRED20 and the mutant M12.

Enzyme	Km (mM)	kcat (s^−1^)	kcat/Km (mM^−1^ s^−1^)
WT	263.04 ± 22.45	80.47 ± 2.06	0.31 ± 0.018
M12	92.15 ± 14.49	387.33 ± 14.01	4.25 ± 0.48

## Data Availability

The data that support the findings of this study are available from the corresponding author upon reasonable request due to privacy or ethical restrictions.

## Supplementary Materials

The following supporting information can be downloaded at https://www.mdpi.com/article/10.3390/molecules29184393/s1. Table S1: The results of the screened mutants; Table S2: The description of the symbols was described in Supplementary Table S1.
