# Supplementary material for: Heterologous Expression of Ketoreductase ChKRED20 Mutant in Pichia pastoris and Bioreductive Production of (R)-1, 3-Butanediol
Source: Molecules. 2024 Sep 16;29(18):4393. doi: 10.3390/molecules29184393 (PMC11433769; doi:10.3390/molecules29184393)
Supplement: Supplementary file 1 [file molecules-29-04393-s001.zip › molecules-3140032-supplementary.pdf]

Supplementary

Supplementary Table S1 The results of the screened mutants

NO	Residue difference relative to WT	Catalytic performance of the enzyme
WT	-	#
1	S153R;	+
2	Q97K;	+
3	S153N;	+
4	A150Y;	+
5	A150R;	+
6	Q97R;	+
7	G94R;	+
8	G94L;	+
9	G94V;	+
10	G94Q;	+
11	G94K;	+
12	A150K;	+
13	S153M;	+
14	S153L;	+
15	S153Q;	+
16	L152Y;	+
17	A46V;I86V;G185C;A187G;	+
18	A46V;I86V;G185C;A187G;S228A;Y239A;	+
19	A46T;	+
20	A46T;G185C;A187G;M235V;	+
21	G185C;A187G;S228A;	+
22	A46T;G185C;A187G;	+
23	A187G;S228A;M2351;Y239A;	+
24	A46T;G185C;A187G;S228A;	+
25	G185C;A187G;Y239A;	+
26	I86V;G185C;A187G;S228A;	+
27	A46V;G185C;A187G;Y239A;	+
28	A46V;I86V;G185C;	+
29	A46T;I86V;G185C;A187G;S228A;	+
30	A46V;G185C;	+
31	A46V;I86V;Y239A;	+
32	A46T;G185C;	+
33	A46T;A187G;	+
34	A46V;G185C;A187G;S228A;	+

35	I86V;S228A;Y239A;	+
36	I86V;G185C;A187G;Y239A;	+
37	A46V;	+
38	A46V;G185C;S228A;M2351;Y239A;	+
39	I86V;G185C;A187G;	+
40	A46T;I86V;G185C;S228A;	+
41	A46V;G185C;A187G;	+
42	A46V;S228A;M2351;	+
43	A46T;I86V;G185C;S228A;Y239A;	+
44	G185C;A187G;	+
45	S228A;M2351;	+
46	A46V;G185C;Y239A;	+
47	A46V;G185C;A187G;S228A;Y239A;	+
48	A187G;S228A;M2351;	+
49	A46T;G185C;S228A;	+
50	A46T;I86V;G185C;A187G;	+
51	A46V;G185C;A187G;S228A;M235V;	+
52	A46V;I86V;G185C;A187G;M235V;	+
53	A46V;I86V;G185C;A187G;S228A;	+
54	S16R;	+
55	S65A;	+
56	I114V;	+
57	M141I;	+
58	S16K;	+
59	138C;	+
60	138W;	+
61	S16N;	+
62	A46T;I86V;Q97K;A150K;G185C;A187G;	++
63	A46T;I86V;Q97K;S153R;G185C;A187G;	++
64	A46T;I86V;A150Q;S153N;G185C;A187G;	++
65	A46T;I86V;Q97K;A150R;G185C;A187G;	++
66	A46T;I86V;A150K;G185C;A187G;	++
67	A46T;I86V;Q97R;A150N;S153Q;G185C;A187G;	++
68	A46T;I86V;Q97R;S153Q;G185C;A187G;	++
69	A46T;I86V;Q97K;S153Q;G185C;A187G;	++
70	A46T;I86V;Q97K;A150Q;S153N;G185C;A187G;	++
71	A46T;I86V;S153Q;G185C;A187G;	++
72	A46T;I86V;A150K;S153N;G185C;A187G;	++
73	A46T;I86V;G185A;A187G;Y239S;	++
74	A46T;I86V;G185A;A187G;Y239D;	++
75	A46T;86V;G185A;A187G;Y239T;	++
76	A46T;I86V;G185A;A187G;Y239P	++
77	H42P;A46T;I86V;G185C;A187G;	++
78	138V;H42K;A46T;I86V;G185C;A187G;	++

79	H42R;A46T;I86V;G185C;A187G;	++
80	H42T;A46T;I86V;G185C;A187G;	++
81	N39S;H42K;A46T;I86V;G185C;A187G;	++
82	I38R;A46T;I86V;G185C;A187G;	++
83	H42K;A46T;I86V;G185C;A187G;	++
84	I38R;H42T;A46T;I86V;G185C;A187G;	++
85	138R;H42S;A46T;I86V;G185C;A187G;	++
86	N39S;A46T;I86V;G185C;A187G;	++
87	A46T;I86V;M141N;G185C;A187G;	++
88	I18V;A46T;I86V;G185C;A187G;	++
89	A46T;86V;M141I;G185C;A187G;	++
90	A46T;I86V;M141T;G185C;A187G;	++
91	H42K;A46T;S65A;I86V;G94L;Q97K;I114V;A150Q;S153N;G185A;A187G;	+++
92	A46T;S65A;I86V;Q97K;A150Q;S153N;G185C;A187G;	+++
93	H42R;A46T;I86V;Q97K;I114V;A150Q;S153N;G185C;A187G;	+++
94	H42K;A46T;S65A;I86V;G94L;Q97K;M141N;A150Q;S153N;G185C;A187G;	+++
95	H42R;A46T;S65A;I86V;Q97K;I114V;A150Q;S153N;G185C;A187G;Y239P;	+++
96	H42K;A46T;I86V;Q97K;I114V;A150Q;S153N;G185C;A187G;	+++
97	H42K;A46T;S65A;I86V;Q97K;A150Q;S153N;G185C;A187G;	+++
98	H42R;A46T;S65A;I86V;G94L;Q97K;I114V;A150Q;S153N;G185C;A187G;Y239P	+++
99	H42R;A46T;S65A;I86V;G94L;Q97K;I114V;M141N;A150Q;S153N;G185C;A187G;	+++
100	H42R;A46T;T641;I86V;G94L;Q97K;I114V;A150Q;S153N;G185C;A187G;Y239P;	+++
101	H42R;A46T;I86V;Q97K;I114V;A150Q;S153N;G185C;A187G;Y239P;	+++
102	H42K;A46T;I86V;G94L;Q97K;I114V;A150Q;S153N;G185C;A187G;	+++
103	H42R;A46T;I86V;G94L;Q97KI114V;A150Q;S153N;G185C;A187G;	+++
104	H42K;A46T;S65A;I86V;G94L;Q97K;A150Q;S153N;G185C;A187G.	+++
105	H42R;A46T;S65A;I86V;Q97K;I114V;A150Q;S153N;G185C;A187G;	+++
106	H42R;A46T;I86V;G94L;Q97K;114V;A150Q;S153N;G185C;A187G;Y239P	+++
107	A46T;I86V;Q97K;114V;A150Q;S153N;G185C;A187G;	+++
108	A46T;S65A;I86V;Q97K;I114V;A150Q;S153N;G185CA187G;	+++
109	A46T;I86V;Q97K;1114S;A150Q;S153N;G185C;A187G	+++
110	A46T;S65T;I86V;Q97K;A150Q;S153N;G185C;A187G;	+++
111	A46T;I86V;G94A;Q97K;A150Q;S153N;G185C;A187G	+++

112	A46T;I86V;Q97K;A150Q;S153N;E173D;G185C;A187G;	+++
113	A46T;I86V;Q97K;A150Q;S153N;E173D;G185CA187G;G244A;	+++
114	A46T;I86V;Q97K;A150Q;S153N;N169H;G185C;A187G;M211S;G244T;;	+++
115	A46T;I86V;193V;Q97K;A150Q;S153N;G185C;A187G;	+++
116	A46T;I86V;Q97K;A150Q;S153N;G185C;A187G;G244P;	+++
117	A46T;I86V;Q97K;A150Q;S153N;E173D;G185C;A187G;;M211N;	+++
118	L20Q;H42K;A46T;I86V;Q97K;I114V;A150Q;S153N;G185C;A187G;	++++
119	G30A;H42K;A46T;I86V;Q97K;I114V;A150Q;S153N;G185C;A187G;	++++
120	H42K;A46T;A52S;I86V;Q97K;I114V;A150Q;S153N;G185C;A187G;	++++
121	H42K;A46T;V78R;I86V;Q97KI114V;A150Q;S153N;G185C;A187G;	++++
122	H42K;A46T;G54N;I86V;Q97K;I114V;A150Q;S153N;G185C;A187G;	++++
123	H42K;A46T;A52H;I86V;Q97K;I114V;A150Q;S153N;G185C;A187G;	++++
124	H42K;A46T;A52G;I86V;Q97KI114V;A150Q;S153N;G185C;A187G;	++++
125	H42K;A46T;180N;86V;Q97K;I114V;A150Q;S153N;G185C;A187G;	++++
126	H42K;A46T;180K;I86V;Q97K;I114V;A150Q;S153N;G185C;A187G;	++++
127	H42K;A46T;I86V;Q97K;G104K;I114V;A150Q;S153N;G185C;A187G;	++++
128	H42K;A46T;I86V;Q97K;G104R;I114V;A150Q;S153N;G185C;A187G;	++++
129	H42K;A46T;I86V;Q97K;G104P;I114V;A150Q;S153N;G185C;A187G;	++++
130	H42K;A46T;I86V;Q97K;G104T;I114V;A150Q;S153N;G185C;A187G;	++++
131	H42K;A46T;I86V;Q97K;G104H;l114V;A150Q;S153N;G185C;A187G;	++++
132	H42K;A46T;I86V;Q97K;I114V;G134K; A150Q;S153N;G185C;A187G;	++++
133	H42K;A46T;I86V;Q97K;I114V;G134H;A150Q;S153N;G185C;187G;	++++
134	H42K;A46T;I86V;Q97K;I114V;G134T;A150Q;S153N;G185C;A187G;	++++
135	H42K;A46T;I86V;Q97K;I114V;G134R;A150Q;S153N;G185C;A187G;	++++
136	H42K;A46T;I86V;Q97K;I114V;A150Q;S153N;G185C;A187G;M201A;	++++

137	H42K;A46T;I86V;Q97K;I114V;A150Q;S153N;G185C;A187G;M201E;	++++
138	H42K;A46T;I86V;Q97K;I114V;A150Q;S153N;G185C;A187G;A204D;	++++
139	H42K;A46T;I86V;Q97K;I114V;A150Q;S153N;G185C;A187G;A204N;	++++
140	H42K;A46T;I86V;Q97K;I114V;A150Q;S153N;G185C;A187G;I206A;	++++
141	L10V;H42K;A46T;I86V;Q97K;I114V;A150Q;S153N;G185C;A187G;	++++
142	V22S;H42K;A46T;I86V;Q97K;I114V;A150Q;S153N;G185C;A187G;	++++
143	V35A;H42K;A46T;I86V;Q97K;I114V;A150Q;S153N;G185C;A187G;	++++
144	H42K;A46T;V60S;86V;Q97K;I114V;A150Q;S153N;G185C;A187G;	++++
145	H42K;A46T;V60M;I86V;Q97K;I114V;A150Q;S153N;G185C;A187G;	++++
146	H42K;A46T;L73M;I86V;Q97K;I114V;A150Q;S153N;G185C;A187G;	++++
147	H42K;A46T;I86V;Q97K;I114V;A142S;A150Q;S153N;G185C;A187G;	++++
148	H42K;A46T;Q53T;I86V;Q97K;I114V;A150Q;S153N;G185C;A187G;	++++
149	H42K;A46T;I86V;Q97K;I114V;A150Q;S153N;G185C;A187G;K216R;	++++
150	H42K;A46T;I86V;Q97K;I114V;A150Q;S153N;G185C;A187G;E218S;	++++
151	H42K;A46T;I86V;Q97K;I114V;A150Q;S153N;G185C;A187G;E190D;	++++
152	H42K;A46T;I86V;Q97K;I114V;A150Q;S153N;V1641;G185C;A187G;	++++
153	H42K;A46T;I86V;Q97K;I114V;A150Q;S153N;V164L;G185C;A187G;	++++
154	H42K;A46T;I86V;Q97K;I114V;A150Q;S153N;V184T;G185C;A187G;	++++
155	H42K;A46T;I86V;Q97K;I114V;A150Q;S153N;G185C;A187G;V224S;	++++
156	H42K;A46T;I86V;Q97K;I114V;A150Q;S153N;G185C;A187G;V224T;	++++
157	H42K;A46T;I86V;Q97K;I114V;Y125F;E126S;A150Q;S153N;G185C;A187G;	++++
158	H42K;A46T;I86V;Q97K;I114V;Y125F;A150Q;S153N;G185C;A187G;	++++
159	H42K;A46T;I86V;Q97K;I114V;E126T;A150Q;S153N;G185C;A187	++++

	G;	
160	H42K;A46T;I86V;Q97KI114V;N140V;A150Q;S153N;G185C;A187G;	++++
161	H42K;A46T;I86V;Q97K;I114V;E126A;A150Q;S153N;G185C;A187G;	++++
162	H42K;A46T;I86V;Q97KI114V;E126S;A150Q;S153N;G185C;A187G;	++++
163	H42K;A46T;I86V;Q97K;I114V;Y125F;E126V;A150Q;S153N;G185C;A187G;	++++
164	H42K;A46T;I86V;Q97K;I114V;A150Q;S153N;E173D;G185C;A187G;	++++
165	H42K;A46T;I86V;G94A;Q97K;I114V;A150Q;S153N;E173D;G185C;A187G;	++++
166 **(M12)**	H42K;A46T;I86V;G94A;Q97K;I114V;A150Q;S153N;E173D;G185C;A187G;Y246N;	++++

Supplementary Table S2 The description of the symbols was described in Supplementary Table S1

**Catalytic performance of the enzyme**	**Description**	**Reaction conditions (loading of each substance in the reaction system)**
#	Conversion≤25%, Reaction time≤24h, ee >99%	[72℃, 2.5 h] Heat-treated enzyme solution 30% (v/v), 4-hydroxy-2-butanone 200 g/L, Isopropanol 50% (v/v), NAD^+ 0.2 g/L
+	Conversion≥50%, Reaction time≤24h, ee >99%	[72℃, 2.5 h] Heat-treated enzyme solution 30% (v/v), 4-hydroxy-2-butanone 200 g/L, Isopropanol 50% (v/v), NAD^+ 0.2 g/L
++	Conversion≥75%, Reaction time≤24h, ee >99%	[72℃, 2.5 h] Heat-treated enzyme solution 20% (v/v), 4-hydroxy-2-butanone 200 g/L, Isopropanol 50% (v/v), NAD^+ 0.2 g/L
+++	Conversion≥85%, Reaction time≤24h, ee >99%	[80℃, 2 h] Heat-treated enzyme solution 10% (v/v), 4-hydroxy-2-butanone 200 g/L, Isopropanol 50% (v/v), NAD^+ 0.1 g/L
++++	Conversion≥95%, Reaction time≤24h, ee >99%	[80℃, 2 h] Heat-treated enzyme solution 8% (v/v), 4-hydroxy-2-butanone 200 g/L, Isopropanol 50% (v/v), NAD^+ 0.1 g/L

The same symbol for different mutants, the conversion rate of those mutants are listed in order from small to large, such as the mutants “NO 118-166”, though their catalytic performance of the enzyme are all in the range of “++++”, but the mutant (NO 166) has a higher conversion rate to the mutant (NO 165).
